# Biochemical characterization of zebrafish Paqr5b

**DOI:** 10.1016/j.bbrep.2025.101994

**Published:** 2025-04-02

**Authors:** Md Sohanur Rahman Sohan, Md Forhad Hossain, Shakhawat Hossain, Yuki Omori, Mohammad Tohidul Amin, Md Ekramul Hasan, Toshinobu Tokumoto

**Affiliations:** a Department of Bioscience, Graduate School of Science and Technology, National University Corporation, Shizuoka University, 836 Ohya, Suruga-ku, Shizuoka, 422-8529, Japan; b Biological Science Course, Department of Science, Graduate School of Integrated Science and Technology, Shizuoka University, 836 Ohya, Suruga-ku, Shizuoka, 422-8529, Japan

**Keywords:** Membrane progestin receptor, paqr5b, Zebrafish, Progesterone, Neurosteroid

## Abstract

Previously, we established a gene knock-out strain of paqr5b in zebrafish and showed that the gene is essential for the formation of neurons in the zebrafish olfactory rosette. The results suggested that Paqr5b might play a role as a receptor for neurosteroids and contribute to the differentiation of olfactory neurons.

In this study, we attempted to express the recombinant zebrafish Paqr5b protein and analyze its affinity for steroids. Full-length zebrafish Paqr5b (zPaqr5b) was expressed in *Pichia pastris* according to the method established for goldfish and human Paqr7. Solubilized zPaqr5b was purified by two column chromatography steps, nickel-nitrilotriacetic acid (Ni-NTA) column and gel column (Sephacryl S-300). The protein fraction showed a binding affinity of Kd = 4.6 nM and Bmax = 0.72 nM for progesterone. The result showed that zPaqr5b was successfully fractionated as the active form. The specificity of zPaqr5b against steroids was then analyzed by steroid binding assay. The zPaqr5b showed specific binding to progesterone as well as to the neurosteroid, allopregnanolone (ALLO). In addition, zPaqr5b showed high affinity for 17α,20β-dehydroxyprogesterone (DHP), a pheromone used to induce sexual behavior. In contrast, it was observed that other steroids, estradiol, testosterone and cortisol, showed no affinity, even when present at high doses. These results suggest that zPaqr5b is responsible for a receptor of progestogenic neurosteroids in the differentiation of neurons in the olfactory rosette (OR) and for a receptor for pheromones in developed neuronal cells in the OR.

## Highlights

• Zebrafish Paqr5b protein was expressed and purified as recombinant protein.
• Progesterone binding affinity of Paqr5b was demonstrated.
• Neurosteroid and pheromone binding activity of Paqr5b was investigated.
• Paqr5b is a possible receptor for neurosteroids and pheromones.

## 1 Introduction

Progestin and adipoQ receptor (PAQR) family, which consists of 11 genes, was identified as a new receptor family of GPCR. Among 11 genes, 5 genes, paqr5 to 9, belong to membrane progesterone receptors (mPRs). Paqr5b is a paralog of the fifth member of *paqr* genes in zebrafish. However, *paqrs* are identified as receptors for maturation-inducing hormone in fish, it is suggested that it is involved in a variety of biological functions in different tissues due to its wide range of tissue distribution. Aberrant expression of these *paqrs* has also been reported in human breast, ovarian, bladder, brain, uterine, and prostate cancers. Recently, *paqr5* has been implicated in the development of kidney cancer [[1], [2], [3]].

In zebrafish, high expression levels of *paqr5b* in olfactory rosette (OR) was reported [4]. Previously, we established a zebrafish *paqr5b* gene knockout line and demonstrated that *paqr5b* is required for neuronal cell formation in the OR [5]. Since olfactory sensory neurons (OSNs) originate from a population of multipotent basal cells [[6], [7], [8]], Paqr5b is thought to be involved in the induction of neuronal differentiation from basal cells. Since it has been shown that progestins are required for the development of dendrites in cerebellar neurons [[9], [10], [11]], it is highly possible that Paqr5b is responsible for inducing neuronal differentiation in OR via progestin stimulation.

Paqr5b is also highly expressed in differentiated neurons and is thought to function as a receptor for odorants. In particular, Paqr5b is a possible receptor for DHP, a fish pheromone [12].

The aim of the present study is to analyze the hormone binding affinity of Paqr5b for progestins, especially neurosteroids. Therefore, we expressed and purified recombinant zPaqr5b. The result of binding assay using recombinant zPaqr5b showed that Paqr5b possesses a broad binding affinity for progestins.

## 2 Materials and Methods

### 2.1 Materials

Steroids such as cortisol, progesterone, testosterone, and 17β-estradiol were purchased from Sigma Aldrich Chemicals located in St. Louis, MO. The compound 17,20β-DHP was purchased from Toronto Research Chemicals in Toronto, Canada. Additional chemicals were purchased from Wako Pure Chemical Industries, Ltd. in Osaka, Japan. Furthermore, we purchased Spectra 3.5 kDa dialysis membranes from Spectra Laboratories Inc. in the United States.

### 2.2 Expression and purification of zebrafish Paqr5b

The expression of zPaqr5b in *Pichia pastoris* was performed according to the previously established protocol [13]. A modified technique for the preparation and purification of zPaqr5b membranes was developed, based on methods originally developed for goldfish and human Paqr7 [13,14].

#### 2.2.1 Vector construction and transformation in *pichia* yeast

**Fig. 1:**
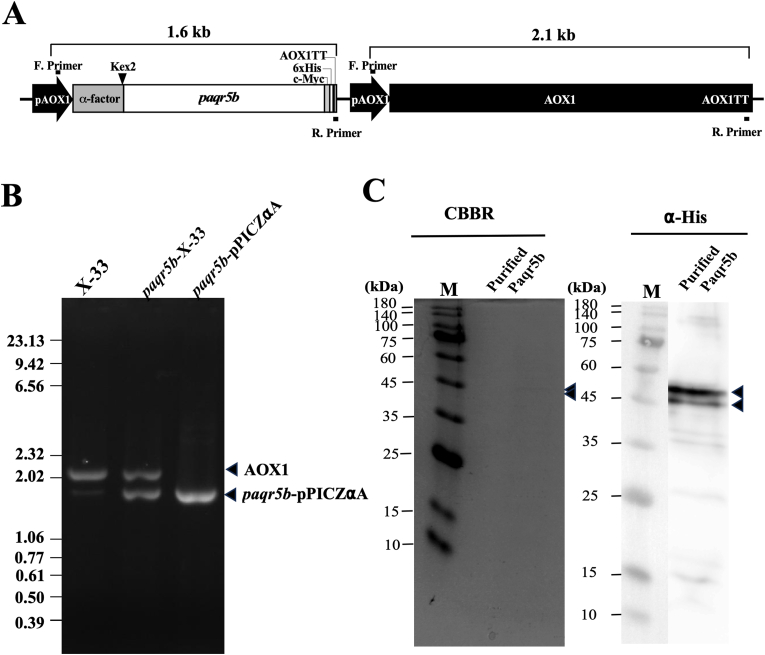
Expression and purification of zPaqr5b in *Pichia pastoris.* (A) A schematic illustration of the zPaqr5b expression cassette, which was integrated into yeast cells for the production of zPaqr5b. This fusion peptide includes zPaqr5b, an α-factor signal sequence, a c-Myc epitope, and a C-terminal histidine tag (6x His), all regulated by the methanol-inducible AOX1 promoter (pAOX1) along with the AOX1 transcription termination region (AOX1 TT). The black bars situated above and below the cassette represent the primer binding sites for the 5′AOX1 (forward primer) and 3′AOX1 (reverse primer), respectively. The AOX1 gene remains part of the expression cassette, which measures 2.1 kbp. (B) The verification of gene insertion was conducted via PCR. DNA fragments were amplified using genomic DNA from non-transformed yeast cells (X-33), genomic DNA from yeast cells transformed with zPaqr5b (zPaqr5b-X-33), or DNA from the transformed vector (zPaqr5b-pPICZαA) as templates. Arrowheads denote the PCR products corresponding to the endogenous AOX1 gene (AOX1) and the inserted zPaqr5b expression cassette (zPaqr5b-pPICZαA). (C) The purified zPaqr5b fraction was analyzed by SDS-PAGE. Protein bands were visualized using Coomassie Brilliant Blue staining (CBBR) or through immunostaining with an anti-His-tag antibody (α-His). Arrowheads indicate the presence of zPaqr5b.

A complete zebrafish Paqr5b (zPaqr5b) CDS was amplified by PCR using primers containing restriction enzyme sites. The resulting cDNA was then digested with *Kpn*I and *Not*I before being cloning into the pPICZαC vector (Fig. 1A). In this *P. pastoris* expression vector, the recombinant zPaqr5b was fused to the pre-pro secretion signal of the *S. cerevisiae* α-factor. The open reading frame (ORF) of the expression cassette was confirmed by DNA sequencing (Fig. 1A). The *P. pastoris* strain X-33 was transformed by electroporation with the zPaqr5b expression construct according to the guidelines outlined in the EasySelect Pichia Expression Kit manual (Invitrogen). Prior to this transformation, the plasmid was linearized with *Pme*I. Electroporation was performed at 1500 V, with a capacitance of 25 μF and resistance of 800 Ω, using a Gene Pulser from Bio-Rad. Recombinant clones were screened on YPD plates (1 % yeast extract, 2 % peptone, 2 % dextrose, 2 % agar) supplemented with 100 μg/mL Zeocin. Genomic integration of the zPaqr5b expression construct was verified by PCR using Ex Taq polymerase (Takara Bio; Siga, Japan) and specific primers (5′ AOX1; 5′-GACTGGTTCCAATTGACAAGC and 3′ AOX; 5′-GCAAATGGCATTCTGACATCC) targeting the AOX1 promoter and terminator sequences surrounding the ORF (Fig. 1A and B). Several Zeocin-resistant clones were evaluated for recombinant protein production, and the top ten clones with the highest expression levels were preserved and stored on MD plates (1.34 % yeast nitrogen base, 4 x 10^-5 % biotin, 2 % dextrose, 1.5 % agar) at 4 °C.

#### 2.2.2 Production of zPaqr5b in *P. pastoris*

Cells were initially cultured in 30 mL of BMGY medium, consisting of 1 % yeast extract, 2 % bactopeptone, 100 mM potassium phosphate (pH 6.0), 1.34 % yeast nitrogen base without amino acids, 4 × 10^−5^ % biotin, and 1 % glycerol, in 200 mL baffled flasks at a temperature of 30 °C with shaking at 250 rpm for a duration of one day. Subsequently, 4 mL of this pre-culture was transferred to inoculate 100 mL of BMGY medium in a 500 mL baffled flask, which was also shaken at 250 rpm at 30 °C for one day. For the induction of zPaqr5b expression using methanol, the cells were harvested via centrifugation and then resuspended in 1 L of BMMY medium, which contained 1 % yeast extract, 2 % bactopeptone, 100 mM potassium phosphate (pH 6.0), 1.34 % yeast nitrogen base without amino acids, 4 × 10^−5^ % biotin, and 0.5 % methanol, to achieve an optical density (OD600) of between 1.0 and 3.0. This medium was then placed in a 2 L baffled flask and incubated at 20 °C for one day. Following this incubation, the cells were pelleted by centrifugation at 3000×*g* for 5 min, rapidly frozen in liquid nitrogen, and subsequently stored at −80 °C until needed.

#### 2.2.3 Membrane preparation and solubilization of membrane proteins

Frozen cell pellets were thawed and then resuspended in an ice-cold lysis buffer consisting of 50 mM sodium phosphate, 1 mM PMSF, 1 mM EDTA, 5 % glycerol, and adjusted to a pH of 7.4. The mixture was subsequently refrozen in the shape of tubules within a stainless-steel chamber designed for cell disruption, utilizing a stainless-steel ball. The cell lysis was performed using a Retsch Ball Mill PM 100 (Verder Scientific Co., Ltd., Haan, Germany), which involved six shaking cycles at a constant speed of 400 rpm for 3 min, interspersed with chilling periods using liquid nitrogen. Following this, the lysed cells were transferred into centrifuge tubes. Cell debris was then separated from the membrane-containing fractions through low-speed centrifugation at 1000×*g* for 7 min at 4 °C. After collecting the supernatant, the pellet was resuspended in ice-cold lysis buffer for an additional round of supernatant extraction. The collected supernatants were pooled together, and the membrane fractions were precipitated through centrifugation at 20,000×*g* for 20 min at 4 °C.

#### 2.2.4 Purification of zPaqr5b

Membrane proteins were extracted by resuspending the pellet in lysis buffer, followed by the addition of n-dodecyl-β-D-maltoside (DDM) to achieve a final concentration of 2 %. After incubating at 4 °C for 1 h, the mixture was centrifuged (20,000×*g*, 4 °C, 20 min) to eliminate insoluble components, leaving the supernatant that contained the solubilized membrane proteins for subsequent purification. To purify recombinant zPaqr5b, the solubilized proteins were introduced to a 100 mL nickel-nitrilotriacetic acid (Ni-NTA) agarose column (Qiagen) (φ4.5 × 20 cm) that had been equilibrated with buffer A (50 mM NaH_2_PO_4_, 300 mM NaCl, 10 mM imidazole, 1 mM PMSF, 0.01 % DDM, 10 % glycerol, pH 6.0). Elution of proteins was performed using a 500 mL gradient of 10–400 mM imidazole in buffer A (50 mM NaH_2_PO_4_, 300 mM NaCl, 40 mM imidazole, pH 6.0 with 1 mM PMSF and 0.01 % DDM, 10 % glycerol). The eluates were divided into 10 mL fractions, and those containing recombinant zPaqr5b, as identified by Western analysis with anti-His tag antibodies, were collected in a dialysis tube and concentrated using sucrose, with less than 5 mL of water removed. In the second purification step, the concentrated sample was applied to a 300 mL Sephacryl S-300 column that had been equilibrated with buffer B (50 mM Tris-HCl and 0.15 M NaCl, pH 8.0, with 1 mM PMSF and 0.01 % DDM). The eluates were again separated into 10 mL fractions and analyzed by Western blot. The purified fractions were pooled, concentrated in a small Ni-NTA column, and stored at −80 °C.

### 2.3 SDS-PAGE and western blot analysis

Proteins were separated using SDS-polyacrylamide gel electrophoresis (SDS-PAGE) on a 12 % polyacrylamide gel under denaturing conditions, following the Laemmli protocol. The proteins were subsequently transferred to Immobilon membranes (Millipore). To block the membranes, a solution of 5 % nonfat dry milk in 20 mM Tris-buffered saline (TBS) at pH 7.6, containing 0.1 % Tween 20 (TTBS), was used for 1–2 h at room temperature. Following this, membranes were incubated with a primary antibody, a monoclonal anti-His-tag antibody (MBL; Nagoya, Japan), which was diluted 1000-fold in TBS buffer. For the secondary antibodies, an anti-mouse peroxidase-conjugated antibody (Invitrogen) was utilized at a dilution of 2000-fold in TBS buffer. Protein detection was achieved through enhanced chemiluminescence with an ECL detection kit (PerkinElmer). The resulting signals were digitized using a CCD camera system, specifically the Luminescent Image Analyzer LAS-4000 mini (Fujifilm).

### *2.4* Radiolabeled ligand binding assays

Progestin receptor binding was assessed in accordance with previously established protocols [15]. At a concentration of 2 nM of [^3^H]-Progesterone (with a specific activity of 40 Ci/mmol), the assay tubes were supplemented with either a 100-fold molar excess of cold progesterone (P4) or test compounds to assess nonspecific and total binding, respectively. The binding reactions were conducted at 4 °C for a duration of 30 min and were subsequently terminated by filtration through Whatman GF/B filters, which had been previously soaked in wash buffer (25 mM HEPES, 10 mM NaCl, 1 mM EDTA, 2.5 % Tween 80, pH 7.4). The filters were then washed three times with 5 mL of wash buffer at 4 °C, and the bound radioactivity was quantified using scintillation counting (Aloka, LSC-8000).

### 2.5 Saturation analyses and scatchard plots

Various concentrations of [^3^H]-P4 (ranging from 0 to 12 nM, with a specific activity of 40 Ci/mmol) were introduced into assay tubes, either with or without a 100-fold molar excess of cold P4, to assess nonspecific and total binding, respectively. Nonlinear and linear regression analyses for saturation curves and Scatchard plots to determine Kd and binding capacity (Bmax) were performed using GraphPad Prism for Macintosh (version 4.0c; www.graphpad.com).

## 3 Results

### 3.1 Expression of recombinant zPaqr5b

To generate zPaqr5b utilizing the yeast *P. pastoris*, the cDNA of zebrafish *paqr5b* was placed between the pre-pro secretion signal of *S. cerevisiae* α-factor and tag sequences (c-Myc and 6 x histidine) within an expression cassette (Fig. 1A). This expression cassette was incorporated into the yeast genome through homologous recombination (see Materials and Methods). The successful integration of the cassette was confirmed via PCR, employing primers that amplified both the gene fusion that was inserted and the native *P. pastoris* AOX1 gene (Fig. 1B). The transcription of the heterologous *paqr5b* gene fusion is regulated by the promoter and terminator regions of the AOX1 gene (Fig. 1A).

Expression of the fusion protein containing an epitope for c-Myc and a C-terminal histidine tag was induced by the addition of 0.5 % methanol to the medium. Western blot analysis of the synthesized recombinant protein, utilizing cell membranes from *P. pastoris* cells containing the expression cassette, revealed two immunoreactive bands when probed with anti-His tag antibodies (Fig. 1C). The observed molecular masses were consistent with the expected molecular masses for zPaqr5b, both with and without the pre-pro α-factor signal peptide, which were 55 kDa and 45 kDa, respectively. These two bands were major bands, detected only after induction by methanol, and were absent in the *P. pastris* extract transformed with empty vector (data not shown). Therefore, we concluded that these two bands were zPaqr5b proteins expressed from the integrated cDNA. As we reported for the goldfish Paqr7 protein, it is suggested that the lower band (45 kDa) corresponds to the digested product of recombinant zPaqr5b at the Kex2 cleavage site (Fig. 1A) [13].

### *3.2* *Purification of solubilized* zPaqr5b *by column chromatographies*

Recombinant zPaqr5b was prepared from large-scale cultures grown in buffered medium containing 0.5 % methanol, from which membrane proteins were solubilized with 2.0 % n-dodecyl-β-D-maltoside (DDM). In the case of zPaqr5b, a higher concentration of DDM was required for solubilization. Recombinant zPaqr5b was purified from the solubilized membrane protein fraction by two-step column chromatography. In the first purification step, the DDM-solubilized membrane proteins were applied to a Ni-NTA column, from which two bands cross-reacting with anti-His tag antibodies were eluted in fractions corresponding to 150–300 mM imidazole in the buffer (Supplementary Figure). The zPaqr5b fractions were then pooled and concentrated to less than 5 ml by sucrose and applied to a gel chromatography column (Sephacryl S-300). The purified proteins could be visualized by protein staining and were reactive with anti-His tag antibodies (Fig. 1C). Among two bands, the high-molecular-weight version was the major one in the purified fraction.

### *3.3* Characterization of steroid binding activity of zPaqr5b

**Fig. 2:**
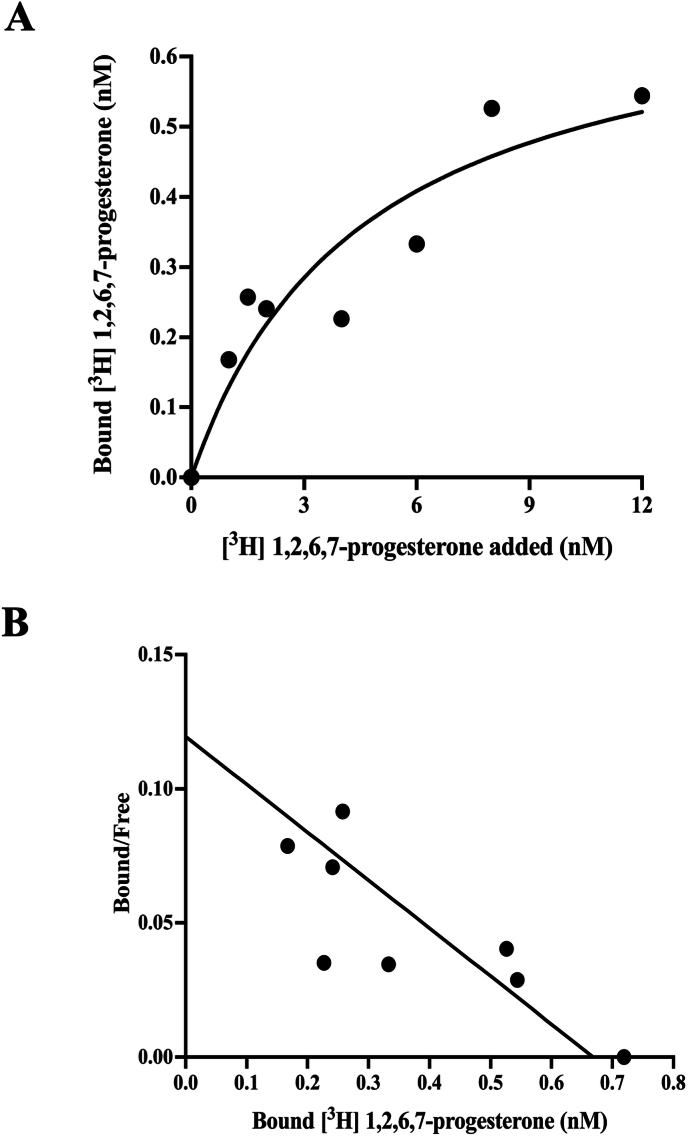
Scatchard plot evaluations of [^3^H]-progesterone interaction with zPaqr5b Saturation curve (A) and Scatchard plots demonstrating specific binding of [^3^H]-progesterone to zPaqr5b (B) across a range of ligand concentrations (0–12 nM).

**Fig. 3:**
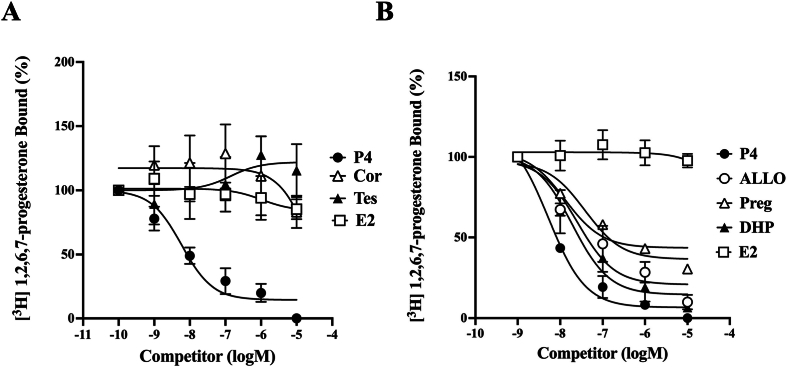
Competition between steroids and neurosteroids for [^3^H]-progesterone binding. (A) Steroid competition. The steroids examined for competition included progesterone (P4), 17β-estradiol (E2), testosterone (Tes), and cortisol (Cor). (B) Neurosteroid competition. The neurosteroids assessed were P4, 17α,20β-dihydroxy-4-pregnen-3-one (DHP), allopregnanolone (ALLO), pregnenolone (Preg), and 17β-estradiol (E2). Samples were incubated with 2 nM [^3^H]-progesterone along with various concentrations of competitors. Each concentration was tested in triplicate, and the mean of these three assays is presented with standard deviation. Competition for [^3^H]-progesterone binding is expressed as a percentage of the maximum specific binding of P4.

The steroid binding activity of zPaqr5b was determined by a filter binding assay using ^3^H-labeled steroids and recombinant proteins as described [15]. Saturation analysis indicated that progesterone binding of zPaqr5b was saturable (Fig. 2A). Scatchard analysis indicated the presence of a single class of high-affinity binding sites (Kd = 4.6 nM) and a limited capacity (Bmax = 0.72 nM) (Fig. 2B). These results suggest that heterologously produced recombinant zPaqr5b is active. Assays for several steroids and progesterone-related compounds were performed to demonstrate the specificity of zPaqr5b (Fig. 3). Among the four steroids, zPaqr5b showed high affinity for progesterone. However, for 17β-estradiol, testosterone and cortisol, zPaqr5b showed significantly lower activity even at high concentrations (Fig. 3A and Table 1). DHP showed competitive binding activity with progesterone. In addition, the neurosteroid allopregnanolone (ALLO) showed high competitive binding activity. Relatively higher binding affinity was detected for pregnenolone, a precursor of steroids produced in the brain. Thus, zPaqr5b showed specific binding activity for all progesterone-related compounds (P4, ALLO, DHP and pregnenolone). The relative binding affinities of steroids and progesterone-related compounds are summarized in Table 1. Although progesterone showed the highest binding affinity, other progestins showed comparable binding affinities. The results suggest that Paqr5b has a broad range of binding affinity for progestins.

**Table 1:** Ranking of steroid binding affinities to zPaqr5b.

Compounds	IC_50_ (nM)	RBA
P4	5.32	100
DHP	19.15	27.76
ALLO	25.17	21.12
Preg	38.27	13.89
Tes	ND	ND
Cor	ND	ND
E2	ND	ND

IC_50_ refers to the concentration of a competitor that results in a 50 % reduction in the binding of [^3^H]-P4. RBA indicates the relative binding affinity expressed as a percentage in comparison to P4. ND signifies that the measurement was not determined, as the displacement was less than 50 % at a concentration of 10^−5^ M.

## 4 Discussion

Previously, we showed that paqr5b is involved in the formation of neuronal cells in the OR, since neuronal cells were absent in paqr5b gene-knockout fish [5]. However, high levels of Paqr5b expression were observed on the surface of developing neuronal cells in the OR. The results suggested that Paqr5b plays the role of odorant receptor in OR [5]. Thus, it is hypothesized that Paqr5b is involved in the formation of the olfactory nerve as a receptor for neurosteroids and that Paqr5b plays a role as a pheromone receptor in the olfactory nerve.

In this study, we succeeded in producing and purifying the zPaqr5b and the specificity of zPaqr5b for steroids was addressed. Recombinant zPaqr5b showed high affinity for progesterone-related steroids, progesterone [16,17], the neurosteroid ALLO [11], and pregnanolone, which is a precursor of the neurosteroid that is highly produced in the brain [18]. ALLO has been reported to prevent Purkinje cell apoptosis by acting on mPRα (Paqr7) in the cerebellum [11]. These results suggest that Paqr5b has a role as a receptor for neurosteroids to induce differentiation of neuronal cells.

DHP is responsible not only for the maturation-inducing steroid, but also for the pheromone that induces reproductive behavior in fish [12]. Pheromone signals should be received by neuronal cells in the OR. Paqr5b is highly expressed on the surface of neuronal cells in the OR. Paqr5b showed a high binding affinity for DHP. The result suggested that Paqr5b can act as a receptor for pheromone, DHP in the developed OR to induce sexual behavior in zebrafish.

In this study, the specificity of zPaqr5b was demonstrated. zPaqr5b showed high affinity for neurosteroids including progesterone. Thus, the result supported the notion that Paqr5b has a role as a receptor for neurosteroids are responsible for neuronal cell differentiation in developing OR. The results also supported the notion that Paqr5b has a role as a receptor for pheromones in differentiated neuronal cells in the OR.

Our recent behavioral study showed that *paqr5b* knockout fish showed no attraction to DHP, in contrast wild-type fish showed positive attraction to DHP. The results showed that wild-type fish are triggered by the signal from DHP. This result supports the idea that Paqr5b is a receptor for a pheromone.

## CRediT authorship contribution statement

**Md Sohanur Rahman Sohan:** Writing – original draft, Methodology, Formal analysis, Data curation, Conceptualization. **Md Forhad Hossain:** Investigation, Formal analysis, Data curation. **Shakhawat Hossain:** Methodology, Formal analysis, Data curation. **Yuki Omori:** Formal analysis, Data curation. **Mohammad Tohidul Amin:** Formal analysis, Data curation. **Md Ekramul Hasan:** Formal analysis, Data curation. **Toshinobu Tokumoto:** Writing – review & editing, Supervision, Resources, Project administration, Methodology, Funding acquisition, Conceptualization.

## Declaration of competing interest

The authors declare that there no conflicts of interest.

## Data Availability

Data will be made available on request.
