# Supplementary material for: Biochemical characterization of zebrafish Paqr5b
Source: Biochem Biophys Rep. 2025 Apr 2;42:101994. doi: 10.1016/j.bbrep.2025.101994 (PMC11999305; doi:10.1016/j.bbrep.2025.101994)

Supplementary Figure 1


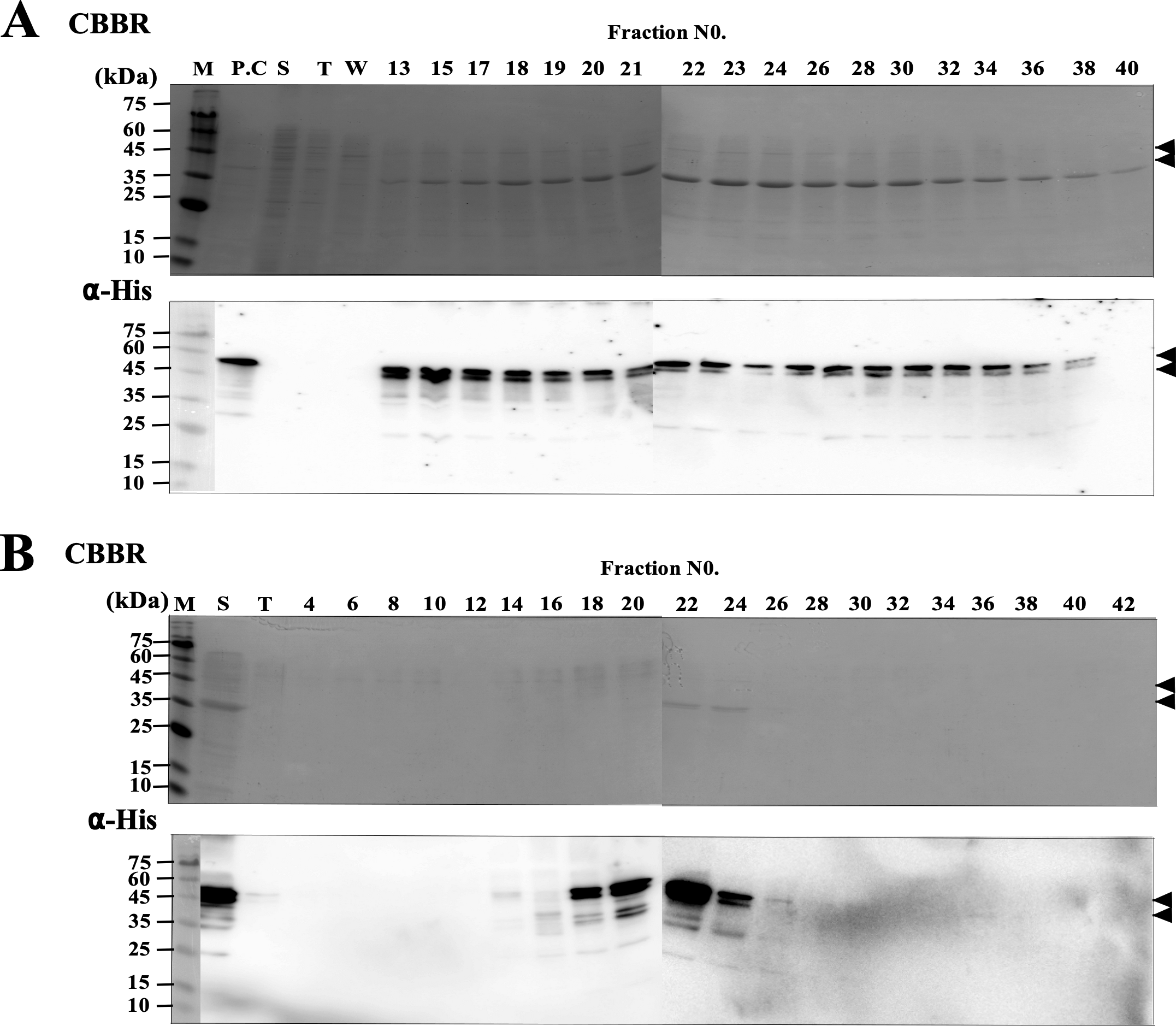


Purification of mPRα protein using Ni-NTA and Sephacryl S-300 gel column chromatography. (A) SDS-PAGE and Western blot analysis of Ni-NTA column chromatography fractions. Fractions 22 to 34 were concentrated and used for the next step. (B) SDS-PAGE and Western blot analysis of fractions from Sephacryl S-300 gel column chromatography. Fractions 14 to 20 were pooled and concentrated as the purified fraction. Protein bands were detected by Coomassie Brilliant Blue staining (CBBR) or immunostained with anti-His (α-His) tag antibody. Arrowheads indicate zPaqr5b.

**Whole uncropped images for Figure 1.**

Whole gel images of CBBR stain and Western blot are indicated with cropped region. Cropped regions are indicated by red squares.


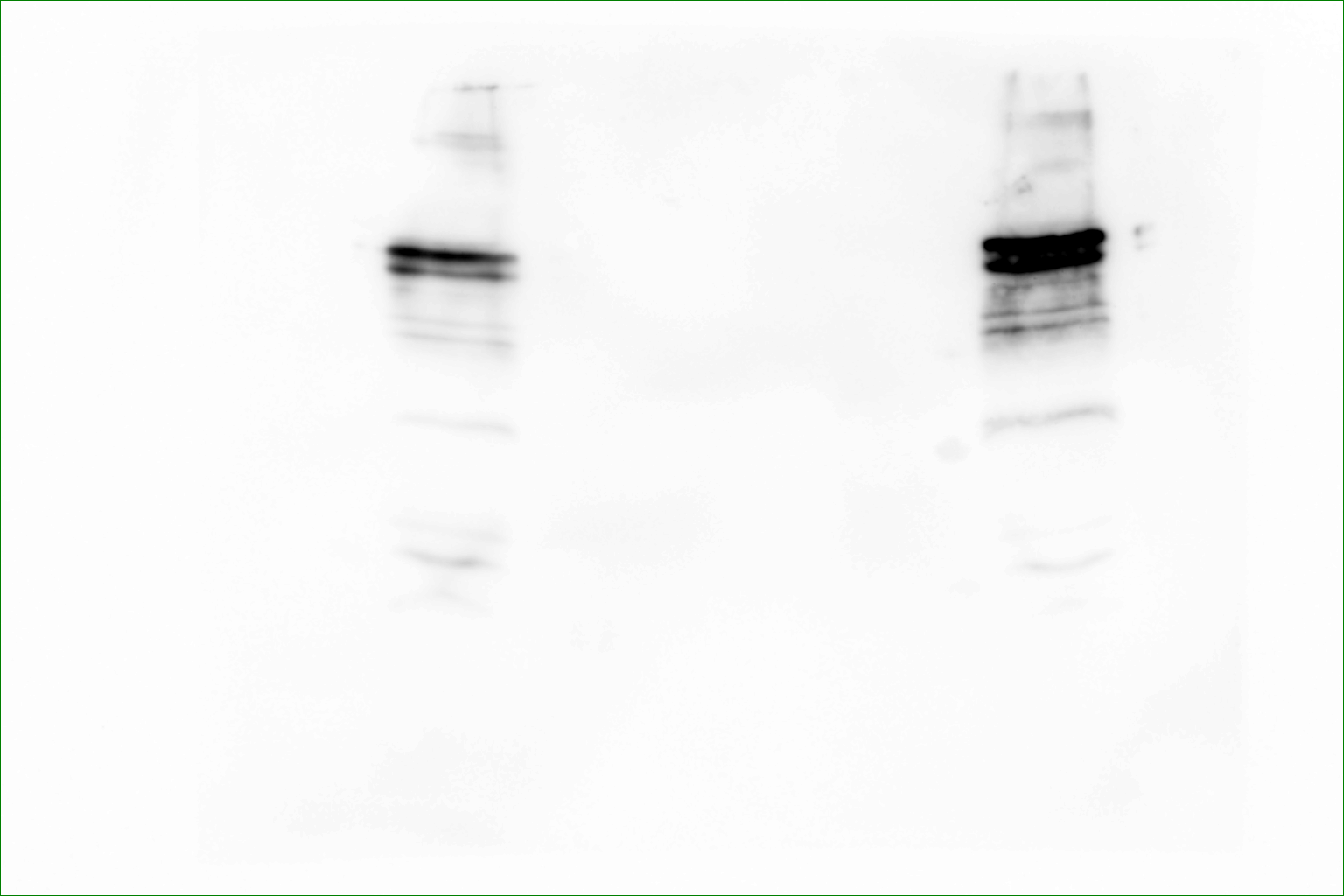

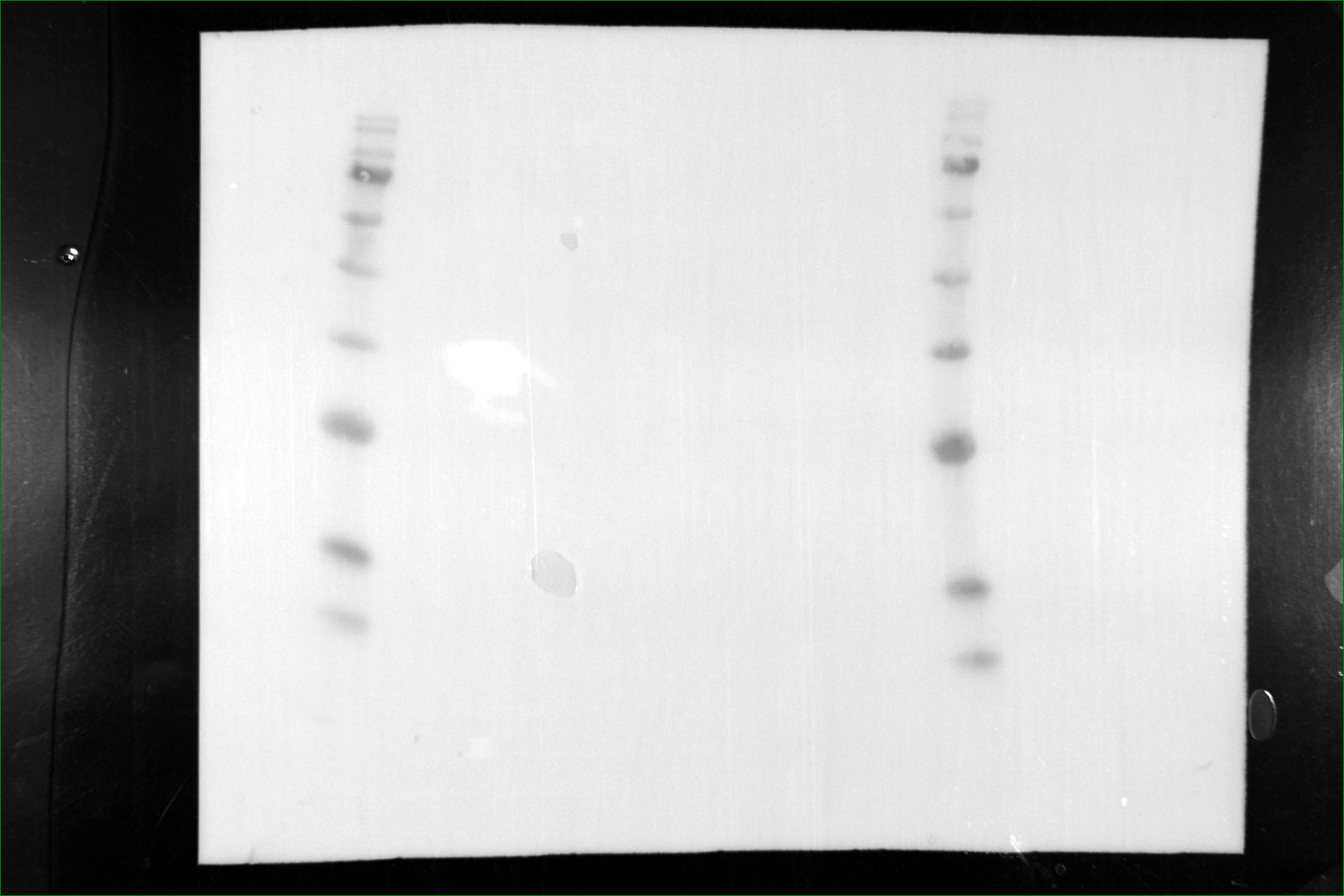

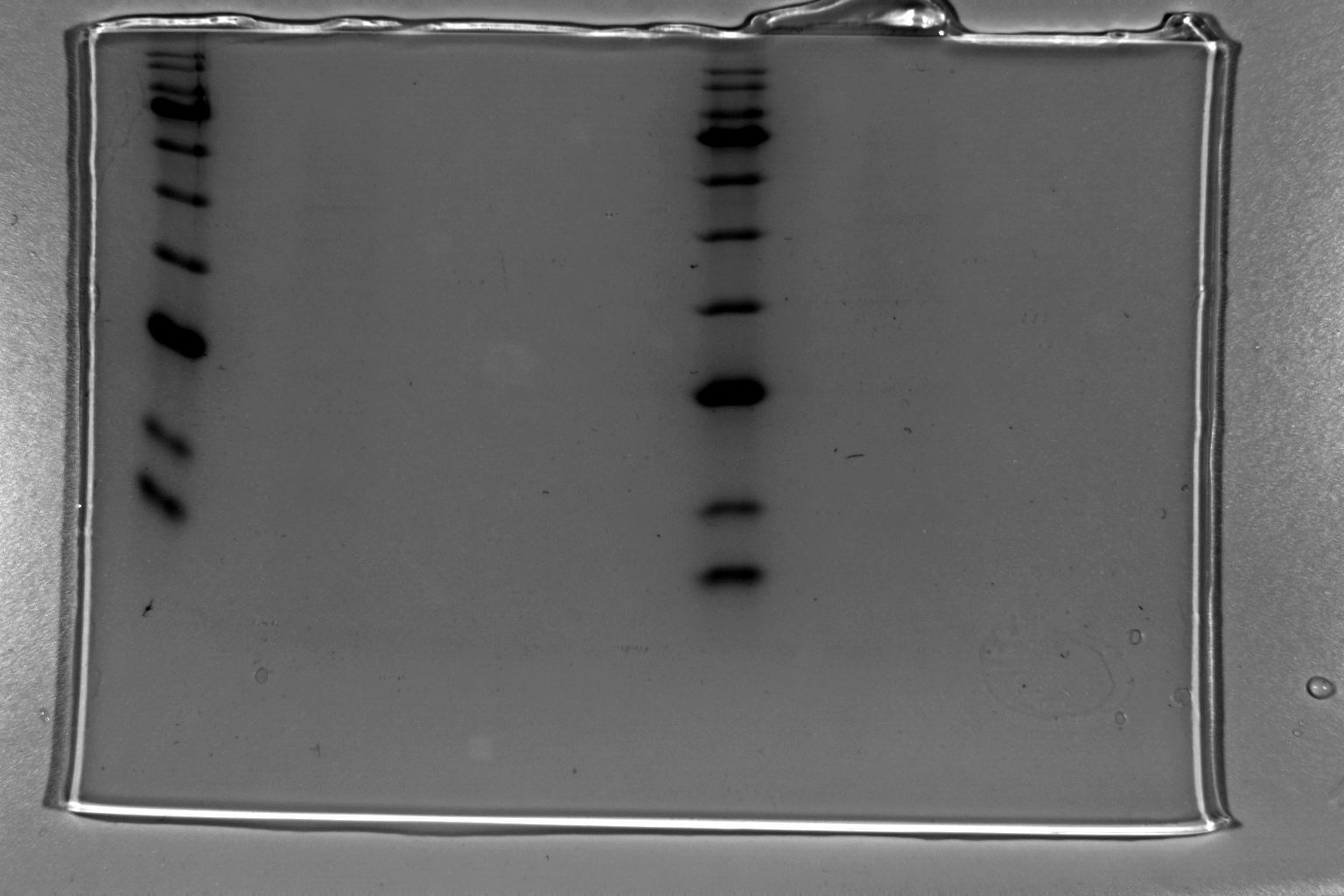

Supplement: Multimedia component 1 [file mmc1.docx]
